# Low Energy Availability with and without a High-Protein Diet Suppresses Bone Formation and Increases Bone Resorption in Men: A Randomized Controlled Pilot Study

**DOI:** 10.3390/nu13030802

**Published:** 2021-02-28

**Authors:** Chaise Murphy, Laura D. Bilek, Karsten Koehler

**Affiliations:** 1 Department of Sport and Health Sciences, Technical University of Munich, 80809 Munich, Germany; chaise.murphy@tum.de; 2 Department of Nutrition and Health Sciences, University of Nebraska-Lincoln, Lincoln, NE 68503, USA; 3 College of Allied Health Professionals, University of Nebraska Medical Center, Omaha, NE 68198, USA; lbilek@unmc.edu

**Keywords:** caloric restriction, aerobic exercise, energy deficit

## Abstract

Suppression of insulin-like growth factor 1 (IGF-1) and leptin secondary to low energy availability (LEA) may contribute to adverse effects on bone health. Whether a high-protein diet attenuates these effects has not been tested. Seven men completed three five-day conditions operationally defined as LEA (15 kcal kg fat-free mass (FFM)^−1^·day^−1^) with low protein (LEA-LP; 0.8 g protein·kg body weight (BW)^−1^), LEA with high protein (LEA-HP; 1.7 g protein·kg BW^−1^) and control (CON; 40 kcal·kg FFM^−1^·day^−1^, 1.7 g protein·kg BW^−1^). In all conditions, participants expended 15 kcal·kg FFM^−1^·day^−1^ during supervised cycling sessions. Serum samples were analyzed for markers of bone turnover, IGF-1 and leptin. The decrease in leptin during LEA-LP (−65.6 ± 4.3%) and LEA-HP (−54.3 ± 16.7%) was greater than during CON (−25.4 ± 11.4%; *p* = 0.02). Decreases in P1NP (*p* = 0.04) and increases in CTX-I (*p* = 0.04) were greater in LEA than in CON, suggesting that LEA shifted bone turnover in favour of bone resorption. No differences were found between LEA-LP and LEA-HP. Thus, five days of LEA disrupted bone turnover, but these changes were not attenuated by a high-protein diet.

## 1. Introduction

Energy availability represents the dietary energy remaining for physiological functions following the deduction of exercise expenditure [1]. At a threshold of 30 kcal·kg fat-free mass (FFM)^−1^·day^−1^ [2], an abundance of hormonal disturbances characterizing low energy availability occurs [1]. In addition to the well-documented suppression of sex hormones [3], key metabolic hormones involved in the regulation of bone metabolism, such as insulin-like growth factor 1 (IGF-1) and leptin, are suppressed by low energy availability [2]. Reductions in IGF-1 secondary to low energy availability have been associated with bone loss [4] and reduced bone mineral density [5] while low leptin levels have been linked to a higher risk of fracture [6].

By definition, energy availability can be lowered through a reduction in energy intake, an increase in exercise energy expenditure or a combination of both. This places individuals with large exercise energy expenditures, such as endurance athletes, at a greater risk of experiencing low energy availability compared to those primarily engaging in training modalities with a lower exercise energy expenditure, such as resistance training [7]. Furthermore, individuals practicing non-weight-bearing exercise modalities have additional risk for adverse bone health outcomes as non-weight-bearing exercise modalities show less benefit to skeletal health than their weight-bearing counterparts [8]. At the intersection of a high exercise energy expenditure and practice of non-weight-bearing exercise are athletes such as cyclists who, indeed, display an increased risk for low energy availability [9] and a high prevalence of low bone mineral density [10] likely originating from this combination of risk factors. Thus, for these and other athletes at risk for experiencing periods of low energy availability, additional measures to mitigate the harmful effects of low energy availability on bone health are needed.

One strategy with underexplored potential is that of dietary protein. During periods of low energy availability, protein requirements, particularly for athletes, are elevated [11]. Increased dietary protein preserves lean mass, which is positively associated with bone mineral density [12]. Indeed, a high-protein diet has been shown to preserve lean mass and bone mineral density similar to an energy balance control during a six-month weight loss intervention [13]. Maintenance of lean mass and bone mineral density during periods of low energy availability in athletes may improve performance capacity and reduced risk for future injury, or even osteoporosis later in life [14,15]. Mechanistically, high protein intakes may exert these protective effects by attenuating reductions in IGF-1 characteristic of low energy availability exposure. This hypothesis comes from the observation that low protein intakes have been shown to suppress IGF-1 even without the presence of energy restriction [16]. 

Therefore, the purpose of our pilot study was to first confirm the effects of low energy availability induced by a combination of dietary energy restriction and exercise energy expenditure on hormones such as IGF-1 and leptin and downstream markers of bone turnover using a non-loading form of aerobic exercise, namely cycling. Previous research examining the impact of low energy availability induced via exercise energy expenditure on bone markers has been limited to the use of weight-bearing running exercise [17,18]. Additionally, we wanted to explore whether increased dietary protein preserves upstream signals from IGF-1 or leptin and blunts the bone turnover marker response during short-term low energy availability. While IGF-1 has been shown to respond to protein restriction independent of energy restriction [16], leptin is tightly linked to energy availability and likely will not respond to increased protein intake without a change in energy availability. Thus, we hypothesize that low energy availability will decrease circulating IGF-1 and leptin as well as increase bone resorption and decrease bone formation. We anticipate that a high-protein diet will attenuate the effects of low energy availability on IGF-1 and bone turnover markers, but not leptin. 

## 2. Materials and Methods

### 2.1. Study Design

The present randomized, single-blind repeated measures crossover pilot study consisted of three five-day conditions (Table 1), a sufficient duration for detecting changes in metabolic hormones and markers of bone turnover in response to low energy availability [18]. Two conditions restricted energy intake to 30 kcal·kg fat-free mass (FFM)^−1^·day^−1^ (LEA). In one LEA condition (LEA low protein; LEA-LP), participants consumed 0.8 g·kg body weight (BW)^−1^·day^−1^ protein in accordance with the recommended daily allowance. The other LEA condition (LEA high protein; LEA-HP) provided participants 1.7 g·kg BW^−1^·day^−1^ protein, an amount reflecting the upper limit of protein recommended for athletes by the American College of Sports Medicine [19] which has been shown to preserve lean mass during energy restriction [20]. Participants also underwent a condition which provided them 55 kcal·kg FFM^−1^·day^−1^ and 1.7 g·kg BW^−1^·day^−1^ protein, operationally defined as the control condition (CON). Participants expended 15 kcal·kg FFM^−1^·day^−1^ in supervised exercise sessions during all conditions. This resulted in a net energy availability after daily exercise of 15 kcal·kg FFM^−1^·day^−1^ in the LEA conditions and 40 kcal·kg FFM^−1^·day^−1^ in the CON condition. These levels of energy availability have induced significant weight loss and maintained weight, respectively, in a similar intervention [21]. Participants were randomly assigned to one of six condition sequences by a random number generator and completed a washout period of at least two weeks between conditions during which they continued habitual exercise and dietary practices. This duration of washout period is slightly longer than what has previously been used (10 days) to recover body weight and metabolic hormones following short-term exposure to an LEA of 15 kcal·kg FFM^−1^·day^−1^ [21]. The study was approved by the University of Nebraska—Lincoln’s Institutional Review Board (IRB#15895; Approved 17 March 2016) and registered at www.clinicaltrials.gov (NCT02945410; accessed on 19 December 2019).

### 2.2. Participants

We conducted the pilot study between 1 September 2016 and 15 January 2018. Participants were recruited from campus and other local recreation sites via flyers, emails to campus sports clubs and social media posts. Participants were nonsmokers between 19 and 30 years old with a normal body fat percentage (<20%) as measured by skinfold measurement (Harpenden CE 0120, Baty, Burgess Hill, UK) and completed ≥4 h of purposeful aerobic exercise per week for six months prior to beginning the study. We selected young participants for the study to ensure participants could recover quickly from the high physical demands of the study while lean participants lose greater amounts of lean mass during weight loss [22], which maximized effect sizes. Recruiting trained participants reduced training effects of the interventions and ensured participants would be able to complete and recover from daily exercise sessions. Compliance to these inclusion criteria was confirmed during an initial screening visit to the laboratory after the informed consent was signed. 

### 2.3. Preliminary Testing

During preliminary testing, participants had their height and weight taken by an electronic stadiometer (222 and 769, SECA, Hamburg, Germany) and their body composition assessed by bioimpedance analysis (Quadscan 4000, BodyStat, Douglas, UK). Participants also completed a graded exercise test on a cycle ergometer (LC6, Monark HB, Vansbro, Sweden) to assess peak oxygen consumption (VO2peak). Participants began cycling at 60 W for 3 min and the intensity was increased by 35 W every 3 min until volitional exhaustion, which required at least 3 of the following: (1) cadence < 60 rpm, (2) respiratory exchange ratio ≥ 1.1, (3) heart rate ≥ 90% of age-predicted maximum (220-age), (4) plateau in oxygen uptake despite increasing workload, (5) rating of perceived exertion ≥ 19. Respiratory data were analyzed by a metabolic cart (QUARK CPET, COSMED, Concord, CA, USA) and used to determine the intensity corresponding to 60% VO2peak.

### 2.4. Diet Preparation

Participants were provided all food consumed during each five-day condition. Diets consisted of an individually tailored combination of clinical products (Ensure Plus; 4.57 g protein·100 kcal^−1^ and Ensure High Protein; 10 g protein·100 kcal^−1^, both Abbott Nutrition, Chicago, IL, USA) and maltodextrin (Tate and Lyle, London, UK). The LEA-LP diet consisted of Ensure Plus providing 0.8 g·kg BW^−1^ protein and maltodextrin added to achieve a caloric intake of 30 kcal·kg FFM^−1^·day^−1^. In the remaining conditions, maltodextrin consumption was matched with LEA-LP and the required amounts of the two clinical products were calculated to obtain 1.7 g·kg BW^−1^·day^−1^ and either 30 kcal·kg FFM^−1^·day^−1^ (LEA-HP) or 55 kcal·kg FFM^−1^·day^−1^ (CON). Participants consumed their maltodextrin during daily exercise bouts dissolved in 800 mL water·hour^−1^ exercise with 1.2 g sodium chloride·L^−1^ to attenuate dehydration and enhance palatability [23]. We supplied a wholly liquid diet from products used in previous interventions [2] to accurately measure intake and blind participants by matching dietary volume between conditions via dilutions with water. Participants were required to consume their food in ≥3 meals spread throughout the day and each participant consumed the same number of meals every day throughout the entire study. During the conditions, participants were permitted to consume non-caloric beverages, but were asked to record consumption of these products.

### 2.5. Supplementation

To mitigate differences in calcium and vitamin D consumption, we supplemented participant intake of these micronutrients throughout the entire study, including washout periods. Calcium and vitamin D provided during each condition were supplemented to make up the difference from the largest amount provided during the study. Supplementation of calcium during washout periods was calculated as the difference between the amount provided within conditions and habitual calcium intake determined using the Brief Calcium Assessment Tool [24]. Vitamin D was supplemented at the maximal amount provided by any condition. Participants were provided all supplements in pill boxes spacing them into 1–3 doses per day depending on number of supplements consumed.

### 2.6. Daily Exercise Prescription

Daily aerobic exercise sessions on the cycle ergometer were calibrated to expend 15 kcal·kg FFM^−1^·day^−1^ at the power output corresponding to 60% of VO2peak achieved during the preliminary graded exercise test. Duration of the daily exercise sessions was calculated by dividing the target energy expenditure of the exercise session by the rate of energy expenditure at the determined power output. Additional exercise and intense physical activity were prohibited. Compliance was measured via a waist-worn accelerometer (ActiLife G3TX+, ActiGraph, Pensacola, FL, USA).

### 2.7. Measurements and Assessments

All measurements were performed in an identical order before (pre) and after (post) each five-day condition. Participants reported to the laboratory between 0700 and 0800 following an overnight fast of at least 12 h. Body weight and composition were measured as reported for preliminary assessments. Then a blood sample was collected from the antecubital vein. Serum aliquots were stored at −80 °C until analysis.

Commercially available assays were used to measure serum concentrations of IGF-1 [R&D Systems, Minneapolis, MN, USA], insulin-like growth factor binding protein-3 (IGFBP-3) [R&D Systems, USA], Leptin [Mediagnost, Reutlingen, Germany], CTX-I [ABClonal, Woburn, MA, USA], P1NP [Cloud Clone, Katy, TX, USA] and sclerostin [Biomedica, Mountain View, CA, USA]. In-house intraassay variabilities for each assay were 3.24% (IGF-1, sensitivity: 0.056 ng/mL), 3.37% (IGFBP-3, sensitivity: 0.14 ng/mL), 1.92% (Leptin, sensitivity: 0.25 μg/L), 7.66% (CTX-I, sensitivity: 0.1 ng/mL), 6.69% (P1NP, sensitivity: 17.71 pg/mL) and 7.30% (sclerostin, sensitivity: 3.17 pmol/L). The IGF-1: IGFBP-3 Ratio (IGFR) was calculated by multiplying the ng/mL concentrations of IGF-1 and IGFBP-3 provided from the assay by 0.13 and 0.036, respectively, to obtain molar concentrations and dividing the molar concentration of IGF-1 by the molar concentration of IGFBP-3 [25].

### 2.8. Statistical Analyses

Changes from pre- to post-condition were expressed in the original units for body composition outcomes and IGFR and percentage changes for markers of bone turnover (P1NP, CTX-I and sclerostin), IGF-1 and leptin. Prior to analysis, all data were examined for outliers, defined as values greater than three standard deviations away from the mean, and assessed for normality using the Shapiro–Wilk test. Following the removal of one outlier in the IGF-1 data, all data were determined to be normally distributed. All outcomes were first analyzed for the effect of LEA by ANOVA. Post hoc, one-sided paired *t*-tests were then performed on hypothesized differences between LEA-LP and LEA-HP. Sample size was determined based on literature reporting changes in IGF-1 following the reduction in energy availability to 10 kcal·kg FFM^−1^·day^−1^ for five days [2]. From this data, we anticipated an effect size of 1.1 and a sample size of *n* = 7 was deemed sufficient to detect differences with a power of 0.80. All statistical analysis was performed using R (R Core Team, Version 3.6). Unless otherwise stated, all data in text and figures are reported as mean ± standard error of the mean (SEM). We defined statistical significance as *p* < 0.05.

## 3. Results

### 3.1. Participant Characteristics and Compliance

Of the 15 participants allocated to an intervention, 10 participants completed at least one condition and seven participants finished all three conditions (Supplementary Figure S1). At baseline, the seven completers were 23.9 ± 1.5 years of age, weighed 86.9 ± 2.9 kg with 13.4 ± 2.0% body fat and had an average VO2peak of 42.6 ± 2.4 mL·kg^−1^ ·min^−1^. Completers did not differ from participants allocated to an intervention but unable to complete all conditions on any of the aforementioned variables (all *t* > 1.28, *p* > 0.20). 

During each condition, completers exercised at an intensity of 124 ± 12 Watts for 115 ± 10 minutes to expend 15 kcal·kg FFM^−1^·day^−1^. All participants attended 100% of their prescribed exercise sessions in each condition. Participants exchanged their empty beverage containers from the previous day for their next days’ meals at each exercise session in addition to completing a dietary intake log of the beverages for each condition. Based on these procedures, dietary compliance was 100%.

### 3.2. Body Weight and Composition

**Figure 1:**
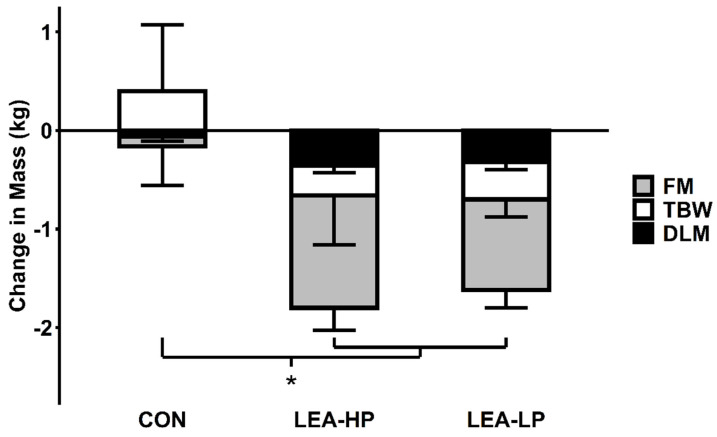
Changes in body composition by condition. Abbreviations: FM, fat mass; TBW, total body water; DLM, dry lean mass; CON, control; LEA-HP, low energy availability with high protein; LEA-LP, low energy availability with low protein. * indicates *p* < 0.05 LEA vs. CON for each compartment.

Completers lost similar amounts of body weight during LEA-HP (−2.27 ± 0.50 kg) and LEA-LP (−2.13 ± 0.30 kg) but not CON (−0.01 ± 0.33 kg; *F* = 19.05, *p* = 0.002). Due to technical difficulties, complete body composition data were only available for five participants (Figure 1). These five completers lost more fat mass (FM) (−1.14 ± 0.23 kg and −0.92 ± 0.18 kg vs. −0.10 ± 0.40 kg; *F* = 8.76, *p* = 0.02) and dry lean mass (DLM) (−0.36 ± 0.07 kg and −0.33 ± 0.07 kg vs. −0.06 ± 0.05 kg; *F* = 10.44, *p* = 0.01) in LEA conditions compared to CON. Losses of FM (mean difference = −0.22, *t* = −1.20, *p* = 0.15) and DLM (mean difference = −0.04, *t* = −0.52, *p* = 0.69) were not significantly different between LEA-HP and LEA-LP.

### 3.3. Leptin, IGF-1 and IGFR

**Figure 2:**
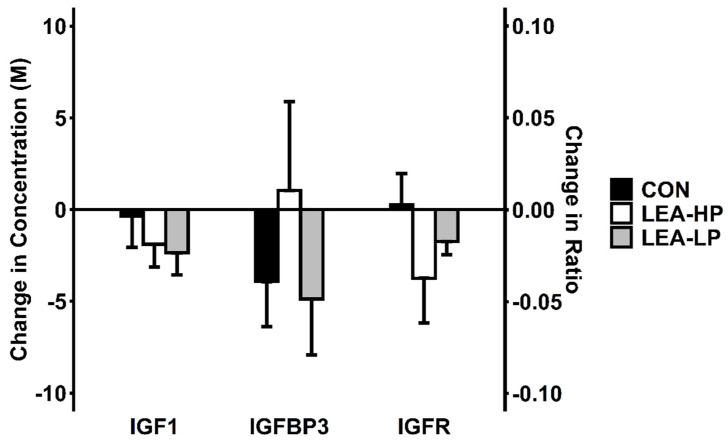
Changes in IGFR and its components by condition. Abbreviations: CON, control; LEA-LP, low energy availability with low protein; LEA-HP, low energy availability with high protein; M, molar concentration; IGF1, insulin-like growth factor-1; IGFBP3, insulin-like growth factor binding protein 3; IGFR, insulin-like growth factor ratio.

Pre- and post-condition measurements for all hormonal outcomes are reported in Table 2. Decreases in leptin were greater during LEA-HP (−54.3 ± 16.7%) and LEA-LP (−65.6 ± 4.3%) conditions than during CON (−25.4 ± 11.4%; *F* = 7.50, *p* = 0.02). The differences in changes in IGF-1 between LEA (HP: −8.1 ± 7.3%; LP −11.8 ± 5.4%) and CON (2.9 ± 9.4%; *F* = 2.42, *p* = 0.14) did not achieve statistical significance (Figure 2). Differences in IGFR changes followed the same pattern as IGF-1 (*F* = 2.37, *p* = 0.15). Due to the lack of difference between LEA and CON conditions, the difference between LEA-HP and LEA-LP was not tested.

### 3.4. Markers of Bone Turnover

**Figure 3:**
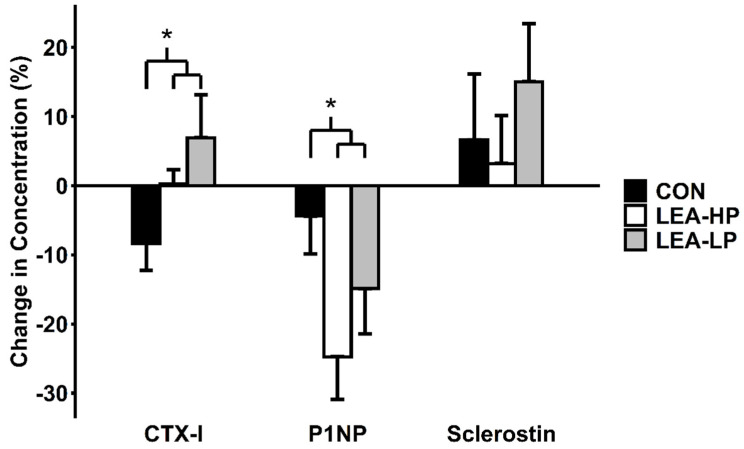
Changes in markers of bone turnover by condition. Abbreviations: CTX-I, type I collagen cross-linked C-telopeptide; P1NP, procollagen type I N-terminal propeptide; CON, control; LEA-LP, low energy availability with low protein; LEA-HP, low energy availability with high protein. * indicates *p* < 0.05 LEA vs. CON.

As shown in Figure 3, P1NP decreased to a greater extent during LEA-HP (−24.8 ± 6.2%) and LEA-LP (−14.9 ± 6.5%) conditions than during CON (−4.4 ± 5.5%; *F* = 4.95, *p* = 0.04). CTX-I increased to a greater extent during LEA-HP (0.2 ± 2.1%) and LEA-LP (6.9 ± 6.2%) conditions than during CON (−8.3 ± 3.9%; *F* = 5.00, *p* = 0.04). However, changes in Sclerostin were not different between LEA and CON (LEA-HP, 3.2 ± 6.9%; LEA-LP, 15.0 ± 8.4%; CON, 6.6 ± 9.5%; *F* = 0.06, *p* = 0.81). None of the changes in the turnover markers above achieved statistically significant differences between LEA-HP and LEA-LP (P1NP, *t* = −1.01, *p* = 0.82; CTX-I, *t* = −0.91, *p* = 0.16; sclerostin, *t* = −0.94, *p* = 0.19, respectively).

## 4. Discussion

The present intervention examined the effects of acute low energy availability exposure on upstream metabolic hormones leptin and IGF-1 as well as downstream markers of bone turnover in men performing daily non-weight-bearing exercise. To our knowledge, the present pilot study is the first controlled low energy availability intervention to explore the ability of a high-protein diet to attenuate the effects of low energy availability on bone turnover. Our results show five days of low energy availability achieved through dietary restriction and daily cycling exercise decreased circulating levels of leptin, but not IGF-1, and reduced bone formation and increased bone resorption. However, the high-protein diet showed a limited ability to blunt these responses.

In agreement with our hypotheses, leptin declined in response to low energy availability. This supports reductions previously observed in lean men [21], sedentary women [2] and a pooled analysis of active men and women [18] in response to low energy availability exposure. Positive associations between leptin and bone mineral density have been widely reported and a growing body of literature supports both direct and indirect mechanisms of action responsible for this observation [26]. Whether reductions in leptin per se are responsible for the shift in bone turnover to favour bone resorption during low energy availability has not been examined. However, we also speculate that leptin makes a poor target for intervention given how robustly it responds to low energy availability, regardless how energy availability is reduced.

In contrast, we were unable to observe a significant effect of low energy availability on IGF-1 or IGFR. Seminal work on the threshold for disruption of hormones showed that IGF-1 decreased in a dose-dependent fashion across 30, 20 and 10 kcal·kg FFM^−1^·day^−1^ in sedentary women [2]. This finding has been supported in a pooled group of men and women by the work of Papageorgiou et al. [18]. However, previous work in lean, trained men similar to our own population did not observe significant changes in IGF-1 at an energy availability of 15 kcal·kg FFM^−1^·day^−1^ [21]. The less consistent findings in men compared to women suggest that IGF-1 may not respond as robustly to low energy availability in men as in women. Potential explanations for this observation include increased peripheral synthesis of IGF-1 by skeletal muscle [27] due to a greater appendicular lean mass in men or a divergent relationship between IGF-1 and estrogen vs. testosterone [28].

Furthermore, a high-protein diet did not beneficially impact the IGF-1 response to low energy availability. We previously showed a single bolus of 30 g whey protein given post-resistance exercise was unable to protect against the decline in IGF-1 observed during low energy availability [29]. We speculated that a more consistent delivery of increased dietary protein (e.g., a high-protein diet) was needed to observe effects of protein on IGF-1. While we were unable to observe such effects in the present study, a one-year weight loss intervention in postmenopausal women did find that a high-protein diet elevated both IGF-1 and IGFBP-3 in addition to improving bone mineral density at several sites [30]. We speculate the relationships between dietary protein, IGF-1 and bone health may be mediated by the preservation of lean mass—which is associated with bone mineral density [12]—and require a sufficient duration for differences in lean mass preservation to appear in order to manifest. However, the aforementioned one-year intervention did not observe any differences in lean mass changes between their protein intakes [30]. Thus, it remains to be determined what role dietary protein plays in moderating changes in IGF-1 and whether this causally influences bone health.

Low energy availability significantly impaired bone formation, indicated by circulating P1NP, and elevated bone resorption, indicated by circulating CTX-I. P1NP has previously been shown to decrease in response to low energy availability induced by daily running exercise [17,18]. This agreement between our results suggests that our choice of a non-weight-bearing exercise modality likely did not contribute to these findings. Previous research has reported a strong correlation (*r* = 0.97) between P1NP and IGF-1 [17]. In the present study, our pre- and post-condition values were only moderately correlated (*r* = 0.66). However, it is promising that we observed a similar P1NP response to LEA in the present study (~15–25%) to that reported in previous research by Zanker and Swaine (15%) [17] and Ihle and Loucks (20–25%) [4]. Though we observed statistically significant differences for CTX-I between LEA and CON, the magnitude of these changes is below reported ranges for intraindividual variability (~10%) [30]. However, our strict control over diet, exercise, physical activity as well as calcium and Vitamin D likely reduced the potential intraindividual variability in the present study.

In the present intervention, consumption of a high-protein diet did not appear to protect against reduced P1NP but showed signs of a protective effect on CTX-I we were not adequately powered to detect. Our observations match that of longer duration interventions which found no effect of a high-protein diet on P1NP [31] and a protective effect on CTX-I [32]. This combination of findings is interesting given that the effects of protein on other tissues, such as lean mass, are often mediated by anabolic and not anti-catabolic effects.

Unlike P1NP and CTX-I, sclerostin did not appear to be impacted by low energy availability in the present intervention. Previously, we reported an increase in sclerostin following just two days of low energy availability and inactivity which was attenuated by performing a bout of resistance exercise on the third day [29]. The absence of a significant increase in the present study suggests that even non-weight-bearing aerobic exercise, when performed daily, may be sufficient to prevent significant elevations in sclerostin during short periods of low energy availability. This is a surprising finding given that sclerostin is produced in response to mechanical unloading and suppressed by mechanical loading [33]. However, sclerostin has previously been shown to respond to both weight-bearing and non-weight-bearing exercise stimuli when both P1NP and CTX-I did not [34]. Thus, it appears that sclerostin may respond more robustly to the exercise stimulus, even during low energy availability, than the standard markers, P1NP and CTX-I, though additional data are needed to support this hypothesis.

Our intervention is one of a limited number of diet and exercise interventions designed to prospectively study the effects of low energy availability exposure. We strictly controlled energy availability through supplying participants with all meals and supervising exercise bouts each day of the intervention. All of this was done to study the effects in men alone. We chose this study population due to the scarcity of research on the effects of low energy availability in men and to help clarify some of the less consistent findings in men compared to women. Albeit small, the sample size (*n* = 7) is similar to previous controlled LEA experiments (*n* = 6–11) [2,17,18,21,29] and our use of a crossover design adequately powered the pilot study to detect changes similar to those seen in previous studies as a result of LEA [2]. Additional research, particularly in larger studies, is still needed to investigate the effects of high-protein diets during LEA. Nonetheless, the present study supports existing low energy availability literature by reinforcing the effects of low energy availability on markers of bone turnover and introducing the potential of high-protein diets to augment the effects of exercise in the context of low energy availability.

## 5. Conclusions

In the present pilot study, low energy availability achieved through a combination of energy restriction and daily cycling exercise reduced circulating levels of leptin, but not IGF-1, in men. Despite this, the combined reduction in bone formation and elevation in bone resorption still signaled a shift in bone turnover favoring resorption. Consuming a high-protein diet during low energy availability did not significantly attenuate these effects. Additional research is needed to further explore the differential responses of IGF-1 to low energy availability between men and women and further investigate the potential of high-protein diets as a strategy to attenuate these deleterious effects.

## Figures and Tables

**Table 1:** Energy and protein characteristics of the three conditions.

Condition	LEA-LP	LEA-HP	CON
**Energy Intake ^1^**	30	30	55
**Exercise Energy Expenditure ^1^**	15	15	15
**Energy Availability ^1^**	15	15	40
**Protein Intake ^2^**	0.8	1.7	1.7

Abbreviations: LEA-LP, low energy availability with low protein; LEA-HP, low energy availability with high protein, CON, control. ^1^ units: kcal·kg FFM^−1^·day^−1^; ^2^ units: g·kg body weight^−1^.

**Table 2:** Biomarker responses to each condition.

Hormone	Condition	Pre	Post	% Change	LEA vs. CON *p* Value
P1NP (μg/L)	LEA-LP	90.9 ± 11.7	77.9 ± 12.2	−14.9 ± 6.5%	0.04
	LEA-HP	85.4 ± 11.8	61.7 ± 7.6	−24.8 ± 6.2%	
	CON	85.0 ± 8.2	80.8 ± 8.7	−4.4 ± 5.5%	
CTX-I (ng/mL)	LEA-LP	1.30 ± 0.16	1.39 ± 0.19	6.9 ± 6.2%	0.04
	LEA-HP	1.22 ± 0.10	1.23 ± 0.10	0.2 ± 2.1%	
	CON	1.47 ± 0.19	1.32 ± 0.14	−8.3 ± 3.9%	
Sclerostin (pmol/L)	LEA-LP	31.4 ± 2.9	36.4 ± 4.7	15.0 ± 8.4%	0.81
	LEA-HP	30.4 ± 3.6	30.8 ± 3.5	3.2 ± 6.9%	
	CON	28.6 ± 5.4	30.3 ± 5.6	6.6 ± 9.5%	
Leptin (μg/L)	LEA-LP	3.28 ± 1.77	1.44 ± 0.89	−65.5 ± 4.4%	0.02
	LEA-HP	2.50 ± 1.21	1.23 ± 0.75	−54.3 ± 16.7%	
	CON	3.03 ± 1.24	2.57 ± 1.51	−25.4 ± 11.4%	
IGF-1 (ng/mL)	LEA-LP	228 ± 30	200 ± 27	−11.8 ± 5.4%	0.14
	LEA-HP	202 ± 29	180 ± 21	−8.1 ± 7.3%	
	CON	225 ± 33	221 ± 20	2.9 ± 9.4%	
IGFBP-3 (ng/mL)	LEA-LP	2418 ± 131	2281 ± 111	−5.2 ± 3.6%	0.61
	LEA-HP	2282 ± 186	2311 ± 80	4.1 ± 7.3%	
	CON	2612 ± 132	2502 ± 117	−3.9 ± 2.7%	
IGFR (no units)	LEA-LP	0.34 ± 0.05	0.32 ± 0.04	-	0.15
	LEA-HP	0.34 ± 0.07	0.28 ± 0.04	-	
	CON	0.32 ± 0.06	0.32 ± 0.04	-	

P1NP, CTX-I, Sclerostin, Leptin (*n* = 7); IGF-1, IGFBP-3, IGFR (*n* = 6). Abbreviations: P1NP, procollagen type I N-terminal propeptide; CTX-I, type I collagen cross-linked C-telopeptide; IGF1, insulin-like growth factor-1; IGFBP3, insulin-like growth factor binding protein 3; IGFR, insulin-like growth factor ratio; LEA-LP, low energy availability with low protein; LEA-HP, low energy availability with high protein; CON, control.

## Data Availability

The data presented in this study are available on request from the corresponding author.

## Supplementary Materials

The following are available online at https://www.mdpi.com/2072-6643/13/3/802/s1, Figure S1: CONSORT 2010 Flow diagram.
