# Supplementary figures and images for: Low Energy Availability with and without a High-Protein Diet Suppresses Bone Formation and Increases Bone Resorption in Men: A Randomized Controlled Pilot Study
Source: Nutrients. 2021 Feb 28;13(3):802. doi: 10.3390/nu13030802 (PMC8000240; doi:10.3390/nu13030802)

**Figure S1: CONSORT 2010 Flow Diagram**

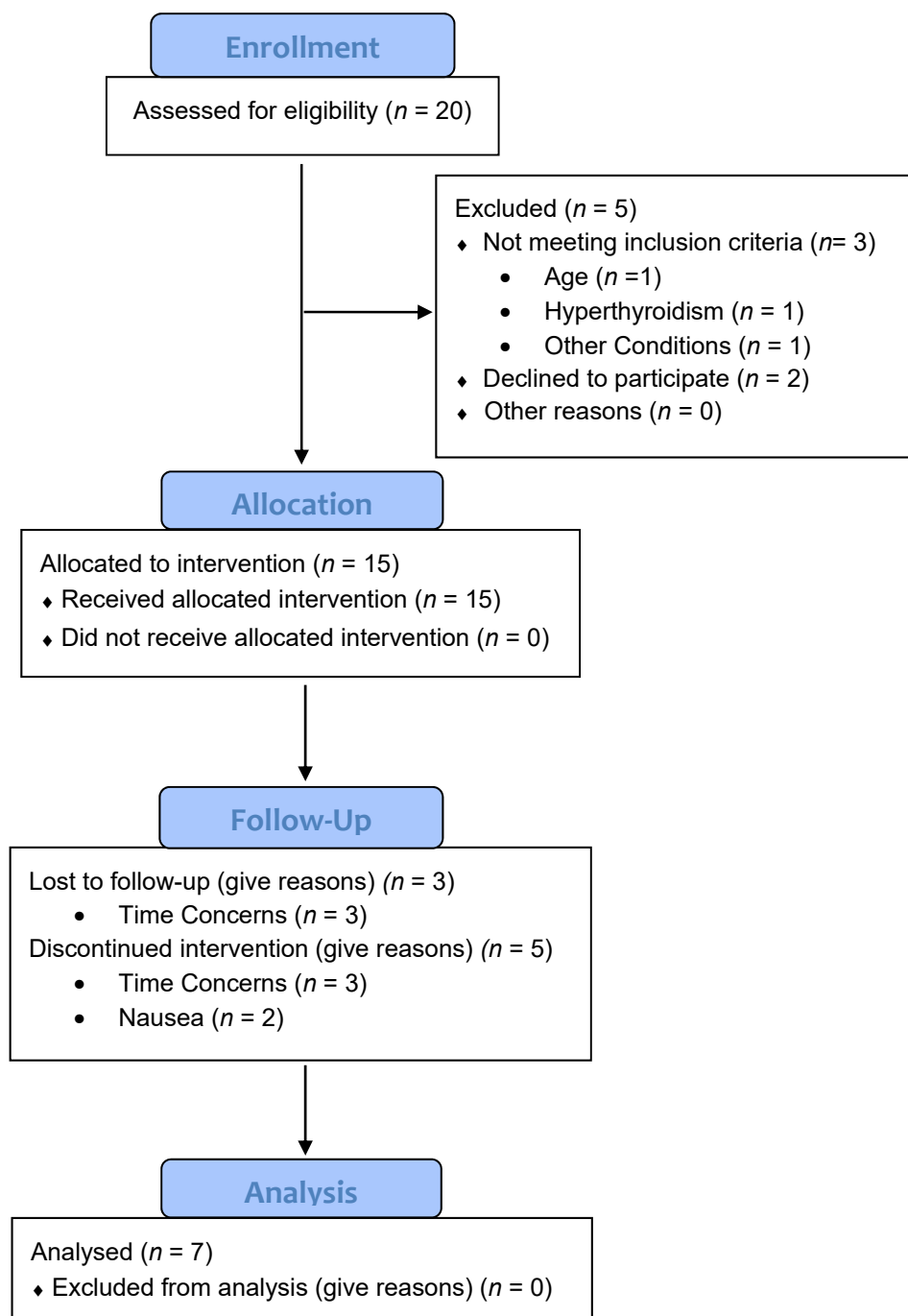

Supplement: Supplementary file 1 [file nutrients-13-00802-s001.pdf]
